# Protocol for a Randomized Controlled Trial Evaluating Mobile Text Messaging to Promote Retention and Adherence to Antiretroviral Therapy for People Living With HIV in Burkina Faso

**DOI:** 10.2196/resprot.5823

**Published:** 2016-08-17

**Authors:** Natascha Wagner, Denis Ouedraogo, Luis Artavia-Mora, Arjun Bedi, Boundia Alexandre Thiombiano

**Affiliations:** ^1^ International Institute of Social Studies Erasmus University Rotterdam The Hage Netherlands; ^2^ Institut du Développement Rural Univsersité Polytechnique de Bobo-Dioulasso Bobo-Dioulasso Burkina Faso

**Keywords:** HIV, PLHIV, mHealth, SMS, RCT, developing countries

## Abstract

**Background:**

Retention in care and adherence to antiretroviral therapy (ART) among people living with human immunodeficiency virus (PLHIV) is a critical challenge in many African countries including Burkina Faso. Delivering text messaging (short message service, SMS) interventions through mobile phones may help facilitate health service delivery and improve patient health. Despite this potential, no evaluations have been delivered for national scale settings to demonstrate the impact of mobile health (mHealth) for PLHIV.

**Objectives:**

This study aims to test the impact of SMS text messaging reminders for PLHIV in Burkina Faso, who are under ART. The evaluation identifies whether patients who receive SMS text messages are more likely to (1) retain in care (measured as a dichotomous variable), (2) adhere to antiretroviral regimens (measured as the number of doses missed in the past 7 days), and (3) experience slower disease progression (measured with T-lymphocytes cells). The second objective is to assess its effects on the frequency of health center visits, physical and psychosocial health, nutrition and whether the type of message (text vs image) and frequency (weekly vs semiweekly) have differential impacts including the possibility of message fatigue over time.

**Methods:**

This 24-month, wide-scale intervention implements a randomized controlled trial (RCT) to evaluate the impact of four variants of a mHealth intervention versus a control group. Our sample comprises adult patients (>15 years of age) undergoing antiretroviral therapy with access to mobile phone services. Multivariate regression analysis will be used to analyze the effect of the intervention on the study population. Data collection is done at baseline and three follow-up waves 6, 12, and 24 months after the intervention starts.

**Results:**

The targeted 3800 patients were recruited between February 2015 and May 2015. But political uncertainty delayed the launch of the intervention until October 2015. Data analysis has not yet started. The first follow-up data collection started in April 2016. To the best of our knowledge, this is the first research that explores the effects of mobile message reminders using a wide-spread sample across an entire nation over a 2-year horizon, especially in a Francophone African country.

**Conclusions:**

We hypothesize that the interventions have a positive impact on retention in care and adherence to ART schemes and that a more sluggish disease progression will be observed in the short run. However, these benefits may fade out in the long run. The study expects to advance the research on how long mHealth interventions remain effective and when fatigue sets in the context of wide-scale interventions. This information will be useful in designing future wide-scale mHealth interventions in developing countries.

## Introduction

### Background

Several factors inhibit retention in care and adherence to antiretroviral therapy (ART) among people living with human immunodeficiency virus (PLHIV). Typical reasons include individual and social obstacles, such as lack of information on treatment procedures, social stigma, discrimination, and competing priorities that prevent patients from considering antiretroviral treatment as a worthwhile investment of time, energy, or resources [1,2]. In developing countries, economic and contextual barriers may further amplify these challenges because patients are resource-constrained and health facilities may not be easily accessible leading to substantial costs in terms of wage losses and travel expenses [3].

Discontinuation of ART regimens is particularly prevalent in Sub-Saharan Africa. Systematic reviews have found that approximately 22.5% of patients discontinue ART within 10 months and 56% are lost to follow-up or death during the first 2 years of the treatment [4,5]. HIV patients are perhaps not fully aware of the long-term consequences of dropping out from ART treatment. Lack of retention in care and adherence to antiretroviral therapy increases HIV viral loads and the probability of transmission, reduces the number of T-lymphocytes cells in the blood (CD4 count)-an indicator of how well the immune system is working and a strong predictor of HIV progression-leads to deterioration in the quality of life and can be responsible for creating virus strains that are resistant to current HIV medication [6-9]. Favorable health outcomes for PLHIV require lifelong compliance with ART programs. At the macro-level the negative side effects of poor compliance with ART can deteriorate the efficiency and efficacy of public health care systems by increasing the burden of the disease and the potential costs of care in the future [10-12].

The World Health Organization (WHO) promotes the use of innovative mobile technologies to overcome barriers that undermine access to health care and the quality of care delivery in resource-poor countries [13]. mHealth is an important element of this approach as it can help alleviate some of the existing obstacles in the delivery of quality care [14-16]. Mobile technologies may help patients undergoing ART to maintain the treatment routine as they provide instant communication unrestricted to location [17,18]. Specifically, the use of text messaging (short message service, SMS) reminders may support PLHIV to take their pills every day, schedule refills of their prescriptions, and assist them through common side-effects. Such reminders are considered to be a low-cost, low-barrier intervention [19]. Especially in resource-constrained developing countries where some patients live far from the health centers and a system of regular home visits by health care providers is not in place [1-3], regular text messages may help patients to remain in care and adhere to their antiretroviral regimens [20-22]. These advantages have promoted the rapid expansion of mHealth projects that aim to improve health outcomes in patients living with HIV and other diseases across the developing world [23].

Despite the possibility of cost-efficient, easy outreach through SMS, recent studies have shown contrasting evidence. While several studies have demonstrated that mobile text message reminders are effective in enhancing adherence to ART programs [24-28] others do not find any effects [29,30]. Research in Kenya demonstrates that 53% of the participants who received weekly SMS reminders achieved adherence of at least 90% during the 12 months of the study [24]. In the control group, only 40% of the participants achieved similar adherence levels. Evidence from a small study in Brazil also suggests that adherence to ART increased due to text message reminders at least during the 4-month study period [28]. In contrast, a 6-month study in Yaoundé, Cameroon, found that standardized motivational mobile text messages did not increase adherence [29]. Likewise, research conducted in some states in India did not find a statistically significant impact of mobile phone reminders on time to virological failure or ART adherence at the end of a 2-year study period [30]. Moreover, we are not aware of any long-term study that has been conducted in a Francophone African country where perceptions, preferences, and health systems are considerably different as compared with Anglophone African countries [31].

### Justification

The background provided above motivates continued research on the impact of mHealth interventions in developing countries. The majority of the existing studies are limited to geographically circumscribed areas such as capital centers, and are based on small sample sizes or on short-time horizons [24-30]. For instance, the Brazilian study carried out a 4-month study with as few as 21 Brazilian female PLHIV [28]. Similarly, the 2 studies in Kenya followed less than 550 participants and included no more than 3 health facilities for a period of 12 months [24,27]. The intervention in Cameroon included 1 hospital and 198 participants for a period of 6 months [29]. While the intervention in India had the longest time horizon of 2 years and a relatively large sample of 631 participants it was based on data from 3 health centers located in only 2 states [30]. Thus, research based on a sample across an entire nation with a longer-term horizon is warranted. The present study is timely as it is based on a wide-spread intervention across Burkina Faso with a 2-year horizon. These design features improve the study’s external validity. Furthermore, the study will enhance our understanding of the extent to which mHealth interventions promote healthy behaviors and support psychosocial wellbeing. Therefore, the study will contribute to an improved understanding of when, why, and for whom mHealth interventions work [32,33].

### Objectives

The main objective of this trial is to determine the impact of four different packages of SMS message reminders to promote HIV patients’ retention and adherence to ART as well as their health outcomes in a large-scale randomized controlled trial (RCT) in a Francophone country, namely Burkina Faso. We hypothesize that patients who receive text messages are encouraged to take their pills and reminded of the importance of ART for their health so that they remain in care longer than those who do not receive text messages. We also anticipate that enhanced retention and adherence will lead to positive health outcomes. Our objectives are (1) to inform best practices for enrolling PLHIV into ART programs and supporting them throughout care, (2) to provide insights on the key obstacles confronting patient retention and adherence to ART, (3) to advise on the most effective application of mobile technology for health interventions, including the short-, medium-, and long-term benefits that can be anticipated, and (4) to encourage long-term patient success with ART by promoting feasible and efficient strategies that may be adopted in resource-constrained settings. Concerning the effectiveness of SMS text messaging reminders, our intervention includes two complementary objectives. First, the study evaluates whether patients may experience fatigue from the SMS text messaging reminders. By carrying out 3 surveys at 6, 12, and 24 months after the launch of the intervention, we aim at determining the optimal period for such a type of mHealth intervention to be efficient. Second, the trial will evaluate the effects of message type (text vs ASCII image) and frequency (weekly versus semi- *weekly)* *,* i.e., the differences in the four treatment arms, on patient outcomes.

## Methods

### Trial Setting

The study is implemented in the Francophone African country of Burkina Faso. The Joint United Nations Programme on HIV/AIDS (UNAIDS) reports that this country suffered a HIV prevalence of 0.9 in 2014 and the total number of PLHIV is estimated to be around 110,000 of which 94,000 are adults and 18,000 are children younger than 15 years [34]. The feminization of HIV is also observed in Burkina Faso since 59 percent of the adult PLHIV are women according to the National Council for the Fight against AIDS and Sexually Transmitted Infections (CNLS-IST) -the national committee in charge of the surveillance and fight against HIV/AIDS. The country provides free anti-retroviral treatment and its provision of HIV care follows WHO’s international guidelines and strategies [35,36]. Burkina Faso’s National Plan to combat HIV includes decentralization policies and multisector participation with the aim to increase the number of PLHIV who enroll at health centers that provide ART therapy and care [37]. Despite these efforts the fight against HIV and for ART adherence and retention remain a concern. Although the actual number of PLHIV enrolled in official files increased from 70,230 in 2013 to 76,342 in 2014, the actual number of patients undergoing active antiretroviral treatment was only 60% (42,145) in 2013 and 61% (46,623) in 2014. Similarly, despite the fact that the number of patients that becomes lost to follow up decreased almost by half from 2013 to 2014 (833 to 443), they are all attributed to fatalities [34]. These figures provide a first indication that adherence and retention to ART cannot be assured in Burkina Faso. And indeed, a survey carried out by UNAIDS in 2012 among 2,800 Burkinabe PLHIV revealed that adherence is perceived as a challenge due to negative side effects from treatment, the time and resources needed to regularly refill the stock of drugs and stigma [38]. The study we aim to implement allows us to assess whether and how retention and adherence may be improved.

### Study Design

The study design rests on a five-arm, randomized, controlled trial with four treatment arms and one control group. With the support of the Burkinabe Ministry of Health and local health centers, HIV patients have been screened and recruited at 80 health care facilities that provide antiretroviral therapy across the 13 regions of Burkina Faso. Using a 7:7:7:7:10 allocation ratio, patients are randomized to one of the five-arms with the control group being slightly oversampled as we introduce two types of control groups; a control group of patients drawn from health centers which do not receive any of the interventions and a spillover-prone control group drawn from health centers which are visited by both treated and untreated patients. A member of the international survey team carried out the randomization in Stata. The team member did not have any interactions with patients. Once patients were randomly allocated, the intervention was launched and the SMS messages are sent. Patients in each of the five groups receive the public standard care but only four groups receive an SMS reminder that varies by the type of message (text versus American standard code for information interchange (ASCII) image) and its frequency (weekly vs semiweekly). The study collaborates with the health care personnel attending to the patients as well as the associations and self-help groups that provide psychosocial support to PLHIV. The former provide medical information and the latter carry out the survey interviews. The health personnel and the enumerators are not informed about the outcome of the random allocation of the participants. Because they do not know which patient is in which group, we do not expect bias. The study will keep track of the patients 6, 12, and 24 months after the intervention is launched.

### Facilities Selection

The investigation will be implemented in health facilities across the 13 regions of Burkina Faso. The health centers are selected from a total of 100 registered facilities that are recorded in the documents of the CNLS-IST as providers of antiretroviral therapy across the 13 health care regions of Burkina Faso [34]. To be eligible to participate in this study, health centers need to fulfill two conditions. First, they need to exist and they need to be functioning. While this sounds like a straightforward requirement it is possible that despite being registered, facilities might have never become operational or have shut down. Second, the health centers need to be willing to collaborate. Specifically, we enrolled a total of 80 health centers with an intended average of 40 patients being recruited per center depending on the eligible population of PLHIV who frequent the health center. To avoid under or overrepresentation of a particular health center, eligible facilities must meet a minimum number of 15 HIV patients and are allowed to enroll a maximum number of 150.

### Participants

#### Eligibility and Informed Consent

The participants need to fulfill five conditions. As an initial step for eligibility, participants must be enrolled in an ART program in 1 of the health care centers collaborating in the study. Second, each patient must provide written informed consent confirming their participation in the study. The informed consent includes signed permission to consult their medical records over the duration of the study. Third, participants must be older than 15 years because the focus of the study is on adult PLHIV. Fourth, participants who have been under ART for less than 4 years are preferred, although, experienced patients are also included. We aim at assessing differential effects for individuals initiating ART versus individuals who have been under treatment for a long time. Because the existing literature suggests that drop-out is highest among those initiating treatment, we place more emphasis on individuals who have recently started ART. Therefore, we aim that at least two-thirds of the sample consists of patients that have been under ART for less than 4 years. We trained and informed the health centers and enumerators about this sampling feature. We requested to be informed (by phone) in the case that patients were included, that have been longer on ART. Thus, from the central level we closely monitored the oversampling of patients, who have been under ART for less than 4 years. Fifth, patients must have reliable access to a mobile phone including a stable network connection. Access to a mobile phone is not a major bottleneck: according to the World Factbook there were 12.5 million subscribers in Burkina Faso in 2014, 68 of 100 inhabitants have a mobile phone, and there are 3 major mobile networks that reach out to the entire country [39,40].

#### Recruitment

The recruitment period lasted for 4 months because patients return to the health centers to refill their stock of antiretroviral medication at different intervals. This time period has been sufficient to reach the target sample size of 3800 individuals.

### Randomization

Randomization was undertaken after the collection of the baseline data. As a first step we randomly identified 8 pure control health centers using the random number generator in Excel. These pure control centers allow us to assess whether there are within health center spillovers from those who receive reminders and those who do not. Across the 72 remaining health centers, we randomly assign individuals to 1 of 5 groups (ie, 1 of 4 treatment packages or the control group of patients who do not receive any text message). Due to the potentially large heterogeneity of patient socioeconomic profiles and variations across health centers, the study applies covariate balancing across multiple baseline covariates to minimize imbalance between treatment groups. To increase statistical power and the precision of the results, participants are ordered along key characteristics and randomized within these ordered blocks. The following characteristics are included in the ordering: gender, age and weight of the participant, duration under antiretroviral treatment, health center identifiers, the reported distance to the health center, CD4 counts, and subjective health rating. Following this 2-step procedure, participants are randomly assigned to 1 of 5 groups (4 of which receive various packages of SMS reminders and a control group, which does not). Thus, there are 2 control groups. One control group consists of individuals who do not receive messages but are affiliated to health centers where others receive messages and a pure control group, which consists of individuals who do not receive messages and are affiliated to the 8 pure control health centers where no one receives a message. The introduction of 2 types of control groups is motivated by a desire to address potential spillovers/contamination between treated persons (those who receive a SMS text message) and patients in the control group (those who don’t receive the SMS text message) but frequent the same health center. We know that medical interventions such as deworming and cancer screenings have spillovers [41,42], similarly voter awareness and cash transfer programs have spillovers to untreated but close populations [43,44]. Having 2 control groups allows us to address whether both treated and untreated patients in the treatment health centers are covered by the intervention [43]. In the analysis, we will introduce a dichotomous variable for control patients from mixed-treatment control sites to assess whether their outcomes differ compared with patients from pure control sites who are unlikely to experience spillovers.

### Intervention

The intervention comprises 4 treatment groups and 1 control group to investigate the impact of different types of SMS text message reminders sent out at different frequencies on patient outcomes. For groups 1 to 4, messages will be sent on a weekly basis. Patients of each of the 4 treatment groups will receive at least 1 text or image message per week. Image messages are used to ensure that the intervention reaches out to people with limited literacy skills and they will be sent as ASCII pictures. Because we do not expect the participants to have smartphones, we use ASCII images that can be displayed on basic mobile phones. The content and frequency of the messages will vary for each of the 4 treatment groups. While the first treatment group will receive only 1 text message per week (low frequency), the second treatment group receives a total of 2 text messages per week (high frequency). Meanwhile, the third group will get 1 text and 1 image message per week and, the fourth group will receive 2 image messages per week. All messages will be sent at 8 am and patients will not be prompted to respond. The impact of the treatments will be assessed in follow-up surveys at 6, 12, and 24 months after the start of the intervention. A detailed identification of the 5 groups, type of interventions, and timings are presented in Table 1.

**Table 1:** Identification of the five intervention groups.

Treatment arms		Intervention (per week)	Timing
**Control group**			
	Eight pure control health centers	No text nor image messages	
	Individuals from the remaining 72 centers randomly allocated to the control group.		
Treatment 1: text-only, low frequency		One text message	Every Monday at 8 am
Treatment 2: text-only, high frequency		Two text messages	Every Monday and Friday at 8 am
Treatment 3: text and image		One text message; one image message	Text: every Monday at 8 am; image: every Friday at 8 am
Treatment 4: image only		One message	Every Monday at 8 am

**Figure 1:**
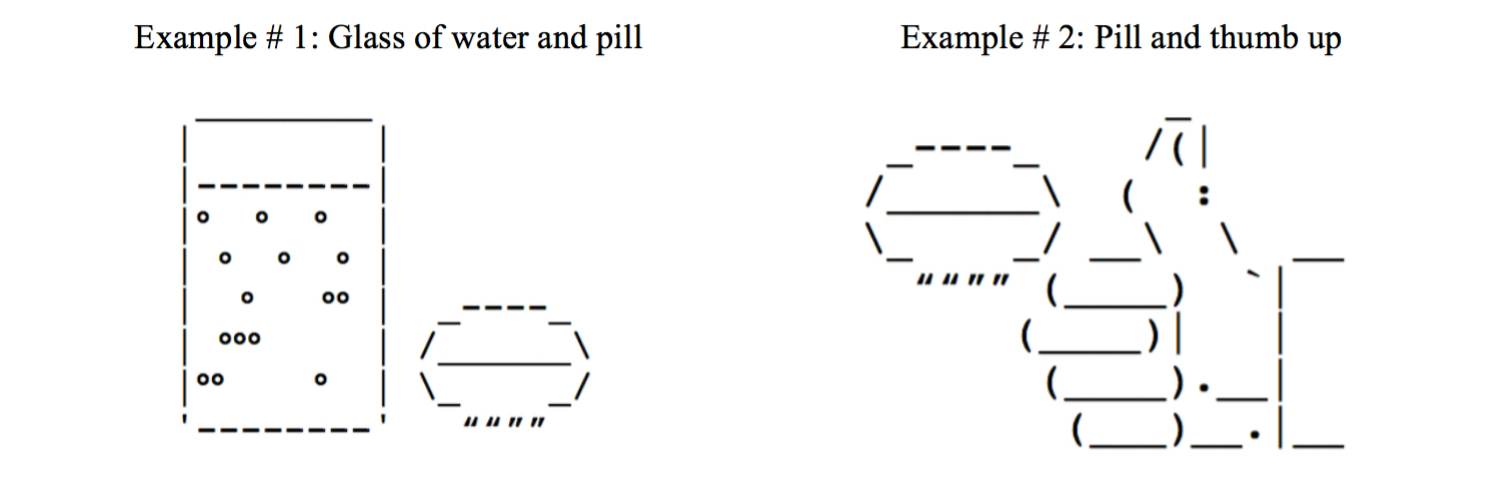
Examples of ASCII images used in the intervention.

Furthermore, text messages are sent in French or 1 of the local languages (Moore, Jula, Gulmancema, Fulfulde, or Dagara). The baseline survey determined the main language of each participant to be used in the text messages. The content of the text messages will vary to ensure that the participants remain curious about the messages over the study period. We are concerned that individuals stop looking at an identical message after some time as they already know the content of the standardized message. Furthermore, we opted for a mix of messages instead of standardized messages due to concerns about exclusively “negative” framing (Textbox 1). However, we acknowledge that this approach does not allow us to systematically compare the implications of standardized messages across different treatment arms. Figure 1 shows two examples of the ASCII images that are sent.

**Examples of the text messages that are sent.**

*Hello. Do not forget to take your pills.*

*Your health is important. Take your pills.*

*You are very important. Do not play with your health. Take your pills.*

*Are you very busy? This is why I remind you to take your pills.*

*Don’t forget that you are strong, unique, funny and blessed. You are needed. This is why I would like to remind you to regularly take your pills.*

Patients in the control group do not receive any text messages but only the standard care. Along with periodic clinical check-ups and treatment counseling, this includes routine monitoring of patient CD4 cells as measure of disease progression. Adherence support and/or additional treatment counseling may also be provided at the community level. This will be assessed during follow-up surveys.

### Sample Size

Power calculations need to account for the stratified nature of the sample. There are 80 treatment centers. We randomly preserve 8 pure control centers to assess spillovers. Within each of the remaining 72 centers, treated and untreated participants are sampled. Thus, for the majority of the centers treatment allocation is not at the level of the center but at the level of the individual. The sample size is calculated with the clustersampsi command of Stata, Version 13 assuming a power of 80% and a significance level of 5%. Furthermore, we impose an intraclass correlation coefficient of 0.015 to quantify the degree to which patients within the same health center are related [45]. We allow for cluster sizes to vary because we know that the size of the eligible population varies across clusters. Thus, we impose a coefficient of variation of cluster sizes of 0.5 (ratio of the standard deviation of cluster sizes to the mean cluster size). Because we collect baseline information we can control for observable characteristics and their correlation with the outcome. We impose a correlation of 0.85. Lastly, because we expect that 35% of the patients will forget to take antiretroviral (ARV) medication from time to time, a target sample size of 3800 PLHIV is needed to obtain a “minimum detectable effect” of an increase in adherence from 65% to 70%. These adherence figures are based on a reported 67% adherence among female PLHIV in Burkina and the government target to bring adherence up to at least 80% [37,38]. As we are conservative about the possibilities of mHealth to raise adherence by 15% the sample was set up in such a way to also identify small gains.

### Duration and Follow-Up Surveys

The intervention will run for a period of 2 years. We will conduct four outcome assessments during baseline as well as 6, 12, and 24 months into the intervention.

### Ethical Concerns

The study faces two ethical concerns. First, we need to access patient medical records to monitor health indicators. We need to gain participant consent and have to ensure that confidentiality is protected during the period of the study. Only the health personnel and the local enumerators will know the individuals. In the dataset, all patient information will be anonymous and only linked to the participant's identification number that is given in the context of the study. No identity information will be disclosed. Second, as evident in the examples provided above, in order to prevent negative social stigma, the reminders do not disclose a participant's seropositive status. Participants will be informed of the possibility that texts could be read by other individuals who have access to their phone and they will have to consent to this risk in order to participate in the study.

All relevant ethical clearance from the national ethics committee has been obtained (N° 2014-12-144).

### Implementation of the Text Message Reminder System

The local telecommunication company EVOLE provides a computerized platform for sending the SMS text message reminders, re-launching text messages if delivery fails, and monitoring message receipt. The messages are sent in bulk and a record is kept of all communication. The computerized platform allows us to verify whether the text and image messages have been sent and that the intervention has been properly implemented. We will also use the monitoring data to analyze the quality of the SMS text message communication.

**Figure 2:**
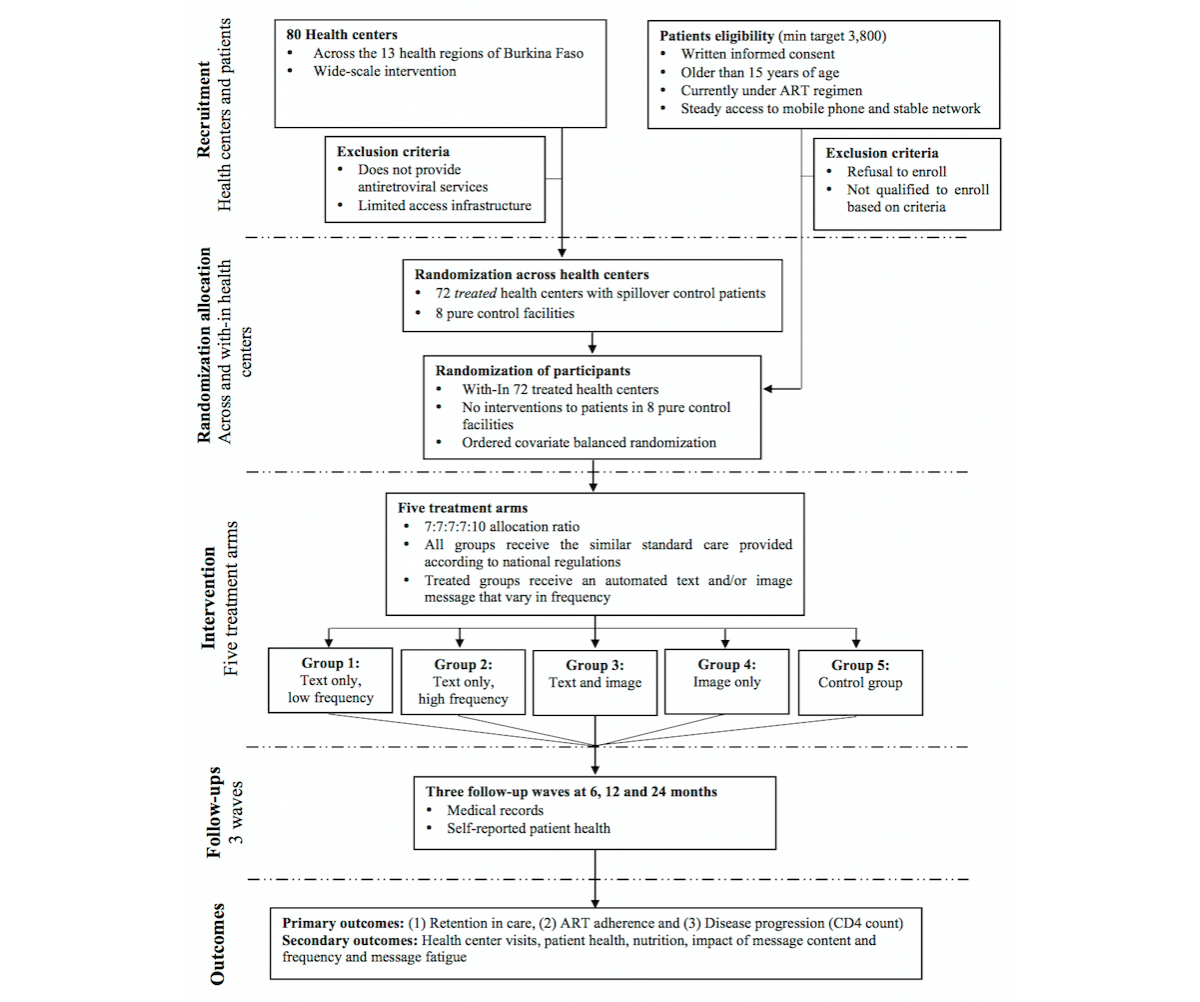
Study flow.

Details about the study design, facility selection, and patient eligibility including the random allocation mechanism, the intervention and the outcome variables are graphically presented in Figure 2.

### Study Measures

#### Primary Outcomes

The study focuses on the measurement of three primary outcomes: retention in care, adherence to ART, and disease progression. Retention is measured by a dichotomous variable-whether a patient remains on ART 6, 12, and 24 months after the intervention has started as compared with baseline [46,47]. Retention could have been conceptualized also as the incidence or number of missed visits during a given reference period [48]. However, remaining in care is in itself an important challenge in Burkina Faso. This is in stark contrast to the situation of PLHIV in developed countries. Therefore, we decided to choose remaining on an ART regimen as our retention measure. Adherence or rather lack of adherence to ART is evaluated as the number of doses missed in the past 7 days, which is a self-reported measure [49-51]. It may have been better to use actual pharmacy dispensing data to calculate a medication possession ratio. However, our field experience suggested that it was not always feasible to obtain detailed and correct information about pharmacy dispensing. Contrary to developed countries where standards across hospitals may be similar, in Burkina Faso we observed considerable differences in the standards and routines across health centers both between regions and within them. Because we assess a wide-scale intervention our study also includes rural and remote areas, which are perhaps even less capable of providing this information. Therefore, we opted for inclusiveness knowing that this implies adjustments in the type of comparable indicators that can be collected and analyzed across health centers [51]. Lastly, disease progression is analyzed using patients’ CD4 counts.

Information on these primary outcomes will be collected using individual questionnaires and patient medical records. Table 2 presents details about the indicators with information about the date of data collection.

**Table 2:** Primary and secondary outcome variables.

Classification		Indicators/measures	Baseline	Follow-ups (months after)		
				6	12	24
**Primary outcomes**						
	Retention in care	Dichotomous measure whether a patient remains on ART	X	X	X	X
	ART adherence	Number of doses missed in the past 7 days	X	X	X	X
	Disease progression	CD4 count	X	X	X	X
**Secondary outcomes**						
	Health center visits	Incidence and number of missed visits		X	X	X
	Patient health	Biomarker: body mass index	X	X	X	X
		Incidence of coinfection	X	X	X	X
		Mortality	X	X	X	X
		Subjective health rating	X	X	X	X
		Measures of mental health		X		X
	Preferences	Risk preference		X		X
		Subjective discount factor		X		X
	Nutrition		X		X	
	Message type and frequency	Comparison of the four interventions		X	X	X
	Message fatigue	Patients' perceptions of the intervention		X	X	X

#### Secondary Outcomes

Information on 5 groups of secondary outcomes will be collected. First, we will obtain information about the incidence and number of missed health center visits. Second, because we are also interested in more general physical and psychosocial health aspects we will gather information about the body mass index, the incidence of coinfections, mortality, as well as subjective and mental health ratings. In addition, we will collect information on the patients’ levels of risk preference and their subjective discount factors. Third, we will also measure nutritional outcomes. Fourth, to assess design effects and the duration of effectiveness of the mHealth intervention, we will gather data on the message type and frequency. The sensitivity to message type (text vs ASCII image) and the frequency (weekly vs semiweekly) of receiving the messages will be measured by comparing the effects of the 4 different interventions on the primary outcomes [24-30]. Finally, message fatigue will be measured using patients’ perceptions and possible changes in the impact on the primary outcome indicators across follow-up survey rounds. The secondary outcomes will be measured 6, 12, and 24 months after commencement of the intervention.

### Analysis Plan

Our analysis will exploit the randomized nature of the intervention to attribute treatment effects. In addition to a simple comparison between the pooled treatments and the control group we will also assess the differential impact of the 4 treatment arms. We will employ multivariate regression models to assess the impact of the interventions on each of the primary outcome variables. For the continuous outcome measure (CD4 count) we plan to employ an Ordinary Least Squares model. For the count data (pill doses missed) and if the continuous outcome measure is skewed, we propose to make use of a Poisson model; for the dichotomous outcome (remaining on ARV regimen), we propose to employ a Logit model. In all models we will control for the clustering of participants within health centers.

We will make use of the coefficient estimates from the 4 treatment arms to establish a preference ordering of the effectiveness of the different types (text vs ASCII image) and frequencies (weekly vs semiweekly) of the SMS text message reminders. We will also estimate quantile regressions to identify which group(s) of participants are most (least) likely to have gained from the interventions. Comprehensive data on patient characteristics collected at baseline will allow us to control for confounding factors and patient heterogeneity. These patient characteristics include age, gender, ethnicity, education, whether the patient is the head of the household, income, and whether the patient works. The simple comparison of outcome variables across groups will be complemented by a difference-in-difference identification strategy where we jointly employ the data from the three follow-up surveys. This analysis will permit us to tease out the differences in health outcomes attributable to each of the 4 interventions while controlling for time-fixed effects.

The data will be collected and coded by trained enumerators and analyzed using the statistical and data analysis program STATA. We will use conventional levels of significance at 1%, 5%, and 10%. Because we identified three primary outcome variables we will make use of an inflated alpha when determining significance levels for each and every outcome separately. We apply this correction because the more significance tests we conduct at α=0.05, the more likely we are to claim that we have a statistically significant result. For a significance level of 5% the Bonferroni inflated alpha is 0.05/3 = 0.015.

We expect missing data to be below 5% and plan to address it using imputation techniques. However, the technique that we do use will depend on the characteristics of the missing information. Our goal is to avoid reducing the sample size without compromising statistical validity.

## Results

Our project finished the recruitment of patients in May 2015. We have recruited the targeted 3800 patients across the 80 health centers and even oversampled by 38 patients. We kept 8 pure control health centers and randomized participants in the remaining 72 treated health centers across the treatment arms and the control group. The intervention started in October 2015 and follow-up data collection has started in April 2016. Analysis of the intervention has not yet started. The project was challenged by the sociopolitical instability in Burkina Faso during 2014, 2015, and early 2016. The initial launch of the study coincided with a coup d’état in Burkina Faso in November 2014. Despite the political turmoil in the country, the study received ethical clearance from the Burkinabe Ethics Committee for Research in Health in December 2014. A second coup d’état in September 2015 further challenged the project. But we could continue with the project and launch the intervention in October 2015. In January 2016, the terrorist attacks in Burkina Faso’s capital Ouagadougou added another layer of uncertainty to the project. Throughout the sociopolitical instabilities in the country the local and the international team managed to keep the project running. We therefore expect that we can successfully complete the project and conduct the intended analysis as outlined in this protocol.

This intervention is funded by 3ie-International Initiative for Impact Evaluation and conducted by the International Institute of Social Studies of Erasmus University of Rotterdam in collaboration with Univsersité Polytechnique de Bobo-Dioulasso.

## Discussion

### Summary

To date, very few rigorous evaluations have examined the impact of SMS text messaging reminders on retention and adherence of PLHIV. Existing studies are based on small samples not delivered widely at a national scale and tend to focus on short time horizons [24-30]. To the best of our knowledge, this study will be the first long-term RCT to assess the effects of a mHealth intervention using an intervention that is delivered at national scale. The study will also assess the effects of message type (text vs ASCII image) and frequency (weekly vs semiweekly), as well as, whether patients experience message fatigue over the course of the 2-year period of the intervention. To assess the impact of this intervention in a multivariate fashion, we will collect information on sociodemographic traits and several diseases-related outcome indicators. The findings of this study will enhance understanding of how interventions using mobile technology can influence HIV care and treatment. The conclusions from this study will contribute to more informed recommendations for mHealth in health care provision and the development of national and international guidelines for mHealth usage. We expect to define when, why, and for whom mHealth interventions may work. The findings may also be applicable to other African countries, which have similar epidemiological HIV profiles and social conditions as in Burkina Faso.

### Limitations

We are not able to control for all possible external factors that may influence the results. These factors include, among others, appropriate supply of medication, political or civil struggles that may influence the functioning of the health system, and other community-level changes during the 2-year intervention period. All these contextual confounders will be documented and considered while interpreting the findings.

### Conclusions

This study aims to contribute to the evidence on mHeath usage to support HIV care delivery in resource-poor settings. We will evaluate the impact of mHealth interventions for care and treatment of PLHIV, and thereby expect to inform strategies to improve health-related outcomes among specific populations. The study expects to advance the research on how long mHealth interventions remain effective in the context of wide-scale interventions in developing countries. We will determine the impact of mHealth in the short-, medium-, and long-term, and thus advance understanding of how mHealth interventions may complement other social and behavioral health interventions in developing countries.

## Abbreviations

ASCII: American standard code for information interchange
ART: antiretroviral therapy
ARV: antiretroviral
CNLS-IST: Conseil national de lutte contre le SIDA et les infections sexuellement transmissibles
CD4: T-lymphocytes cells
HIV: human immunodeficiency virus
UNAIDS: United Nations Programme on HIV/AIDS
PLHIV: people living with HIV
RCT: randomized controlled trials
SMS: short message service
WHO: World Health Organization
